# The European Response to COVID-19: From Regulatory Emulation to Regulatory Coordination?

**DOI:** 10.1017/err.2020.44

**Published:** 2020-04-28

**Authors:** Alberto ALEMANNO

**Affiliations:** * Jean Monnet Professor of European Union Law, HEC Paris, Editor-in-Chief of the *European Journal of Risk Regulation* (EJRR) and Editor of the EJRR Special Issue “Taming COVID-19 by Regulation”; email: alemanno@hec.fr.

## Abstract

Due to its borderless nature, COVID-19 has been a matter of common European interest since its very first detection on the continent. Yet this pandemic outbreak has largely been handled as an essentially national matter. Member States adopted their own different, uncoordinated and at times competing national responses according to their distinctive risk analysis frameworks, with little regard^1^ for the scientific and management advice provided by the European Union (EU), notably its dedicated legal framework for action on cross-border health threats.^2^ To justify such an outcome as the inevitable consequence of the EU’s limited competence in public health is a well-rehearsed yet largely inaccurate argument^3^ that calls for closer scrutiny.

Due to its borderless nature, COVID-19 has been a matter of common European interest since its very first detection on the continent. Yet this pandemic outbreak has largely been handled as an essentially national matter. Member States adopted their own different, uncoordinated and at times competing national responses according to their distinctive risk analysis frameworks, with little regard^1^ for the scientific and management advice provided by the European Union (EU), notably its dedicated legal framework for action on cross-border health threats.^2^ To justify such an outcome as the inevitable consequence of the EU’s limited competence in public health is a well-rehearsed yet largely inaccurate argument^3^ that calls for closer scrutiny.

This article makes a first attempt at unpacking how such fragmented, uncoordinated but ultimately converging national responses to COVID-19 came into being under the EU legal order. To do so, it systematises the European response into separate stages. Phase 1 – the emergency – has been characterised by the adoption of national emergency risk management measures that, albeit country specific, were inspired by a common objective of pandemic *suppression* (ie to reduce disease transmission and thereby diminish pressure on health services) under the by now well-known “flatten the curve” imperative. Phase 2 – the lifting – is about the attempt at relaxing some of the national risk responses in a coordinated fashion to avoid creating negative spill-overs or distortions – be they sanitary and/or financial – across the EU. Lastly, this article strives to define, and possibly predict, the regulatory policy framework that might be governing the next phases of the European risk management response to this pandemic as they will emerge from a widely undefined yet inescapable dialectic between the EU and its Member States.

## I. Phase 1: from divergence to convergence through regulatory emulation

Caught by surprise by the spread of the virus across the continent,^4^ national and EU political leaders found themselves overtaken by an emergency situation. Yet, a pandemic disease of these proportions not only had been predicted,^5^ but also had been identified in the preceding weeks as a high-impact, imminent threat to the European region (and the rest of the world) by both the World Health Organization (WHO) and the European Centre for Disease Prevention and Control (ECDC).^6^

Due to such unpreparedness combined with the gradual time–spatial transmission of COVID-19, the outbreak prompted initially different risk regulatory responses across EU Member States. However, despite some hesitation and a few controversial outliers, notably the UK and Sweden,^7^ Member States have swiftly converged on a very similar risk response: suppression as opposed to mere mitigation of the virus.^8^ Suppression entails the reduction of the reproduction number (the average number of secondary cases each case generates), R, to below 1.^9^

**Figure 1.**
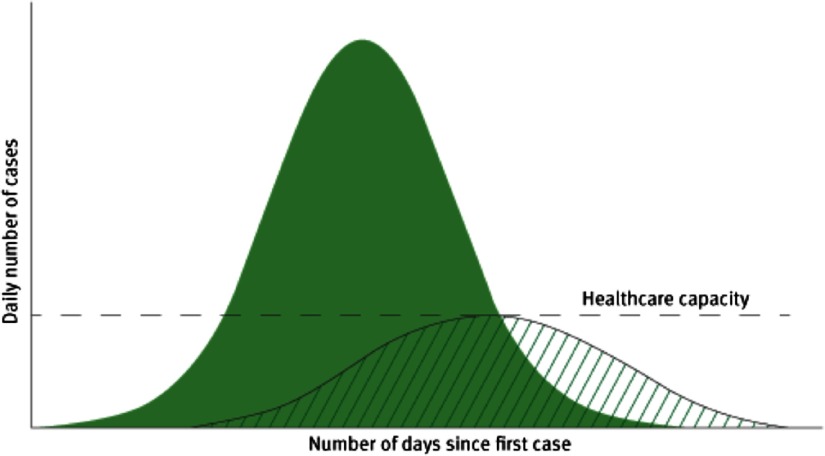
Illustration of the objectives of social distancing measures to reduce and delay the peak of the epidemic and protect healthcare capacity.

Suppression pursues a dual aim (Figure 1):

(i) To reduce disease transmission;
(ii) Thereby reducing pressure on health services.

As Member States gradually converged on suppression as the ultimate goal of their individual and collective interventions, they embraced “social distancing” as the preferred non-pharmaceutical approach (NPA) to attain their goal.

The chosen NPA essentially translates into a set of specific “social distancing measures”, operationalised through regulatory requirements and/or recommendations. Social distancing measures can be listed from the least intrusive and targeted to the most wide in scope: (1) isolation of cases (infected); (2) quarantine of contacts (healthy individuals); (3) stay-at-home recommendations; (4) school closures; (5) workplace closures; (6) measures for special populations, such as long-term health facilities or jails; (7) mass gathering cancellations; (8) cordon sanitaire/mandatory quarantine of a building or residential area(s); and ultimately (9) social distancing of entire population. In the latter case, all households reduce contact outside the household, school or workplace, as they cannot leave their house unless to buy essential products or other contingencies strictly defined.^10^

As a result, virtually all Member States, albeit affected to different degrees by the virus, required some forms of social distancing of the entire population. This, however, occurred through a different country-by-country “regulatory mix” of social distancing measures, be they the prohibition of public gatherings or workplace and school closures (total or partial), as well as the introduction of travel restrictions^11^ – both for intra-state and intra-EU mobility. In addition, virtually all member states have been combining these mandatory interventions with other NPAs – mostly via voluntary measures^12^ – such as:

(i) Personal protective measures (eg hand and respiratory hygiene, cough etiquette and use of respirators or facemasks); and
(ii) Environmental measures (eg routine cleaning of frequently used surfaces, clothes and objects, minimising the sharing of objects and ensuring appropriate ventilation).

**Figure 2.**
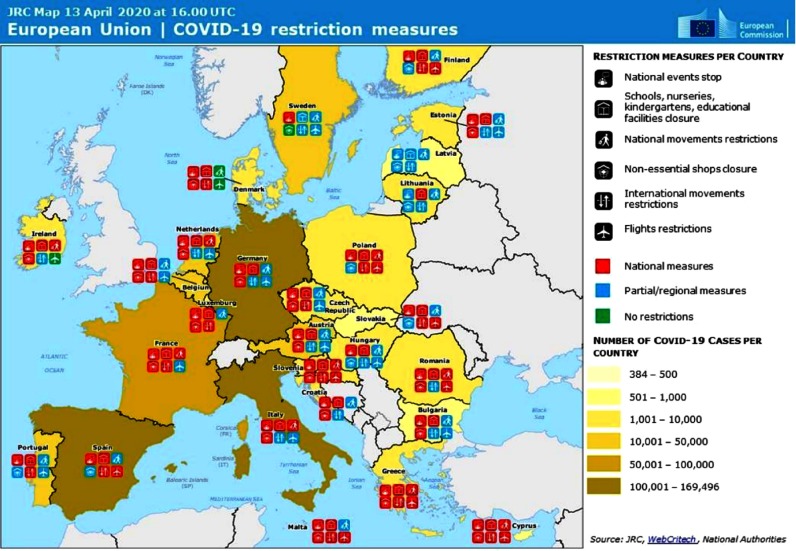
Illustration of the national risk management responses to COVID-19 based on a common taxonomy.

Ultimately, all Member States have prohibited public gatherings, closed (totally or partially) schools and – albeit to different degrees – home confined their populations and introduced some border/travel restrictions. In addition to the adoption of dedicated national COVID-19 risk management policies, more than half of the Member States have proclaimed states of emergency (Figure 2).^13^

Phase 1 of COVID-19 in the EU was therefore initially characterised by regulatory variation across Member States, but was promptly interrupted by spontaneous regulatory convergence. In this initial phase, albeit affected to different degrees by the virus, Member States realised that, given the inherent uncertainty surrounding the nature and spread of the disease, the most responsible course of action was to take the most risk-averse position. Suddenly, the incentives for governments were no longer to identify the most effective course of action based on available context-specific costs and benefits, but rather to emulate the risk responses of the countries hit first by the pandemic. This occurred even when they harboured doubts as to whether they were the best approaches possible. This phenomenon of rapid regulatory convergence prompted by an emergency and an inherent path-dependence dynamic has been defined “copycat coronavirus policies”.^14^ It fundamentally boils down to an instance of “regulatory emulation”, driven by a combination of unmeasurable uncertainty and public pressure in a situation of emergency.^15^

It is worth noticing that such an initial emulation and therefore convergence among EU Member States’ national responses occurred spontaneously, with no direct role played by the EU and its cross-border health emergency coordination mechanisms.

However, while emulation has led to a relatively rapid convergence of phase 1 national responses towards a common approach of generalised, indiscriminate social distancing aimed at suppression, these national responses translated into different mixes of regulatory interventions whose enforcement level significantly varied across Member States.

This explains why many of these national responses to COVID-19 raise, due to their inherent cross-border spill-overs, major concerns under EU law. Yet, surprisingly, only a few of them have been timidly denounced – at the time of writing – by the EU Commission as the Guardian of the Treaties.^16^ It is a legitimate question to ask why and for how long the European Commission could continue to de facto suspend the enforcement of EU law across policy areas in the name of the unfolding emergency. This appeared all the more urgent given the progressive entry into phase 2 of the European response, which is instead characterised by the gradual or less gradual lifting of the national risk responses.

Before providing an account and close legal examination of phase 2 of the EU response to COVID-19, let us turn to the legal framework that enabled the emergence of such “copycat coronavirus policies” during the emergency phase (phase 1) of the outbreak.

## II. The genesis and legality of European “copycat coronavirus policies”

While in phase 1 the national risk responses eventually rallied around a common goal – that of suppressing the disease through social distancing – this occurred more through a spontaneous regulatory emulation process than deliberate design. The question is then why and how this occurred in the first place. In other words, why was the EU unable to coordinate the national risk responses to COVID-19 through its public health powers?

The emergence of uncoordinated national responses should not be seen as the inevitable consequence of the EU’s limited competence in public health.

True, while the Treaty mandates that a high level of human health protection be guaranteed in all EU policies, Article 168(5) of the Treaty on the Functioning of the European Union (TFEU) explicitly excludes the possibility of the EU adopting public health harmonising measures or organising and delivering health services and medical care on this basis. However, this very same provision entrusts the EU to play *inter alia* a complementing, supporting role by coordinating Member States – which maintain the main responsibility for public health – in the “fight against … serious cross-border threats to health”, and “also adopt incentive measures designed to … combat the major cross-border health scourges”.

To protect citizens from such threats – notably, by “improving surveillance and preparedness for epidemics” – is one of the three strategic objectives of the current EU Health Policy.^17^ This alone suggests that the EU public health competence should not be read in isolation, but in conjunction with other health-related legal bases, such as *inter alia* disaster protection under Article 196 TFEU.^18^

Since the early 1990s, the EU set up a network to ensure the epidemiological surveillance of communicable diseases^19^ and an Early Warning Response System for the prevention and control of these diseases. Following the SARS and H1N1 outbreaks, this network was upgraded to a fully-fledged legal framework for EU action on health emergencies – the Cross-border Health Threats Decision.^20^ This is coordinated by the Health Security Committee (HSC),^21^ which has been in existence since 2001 and is made up of representatives from the ministries of health. The HSC builds upon the scientific input of the ECDC. In the case of COVID-19, an ad hoc advisory expert panel – composed of epidemiologists and virologists from different Member States and chaired by the EU Commission President – has been set up to formulate “EU guidelines on science-based and coordinated risk management measures”.^22^

Following an alert notification,^23^ the Cross-border Health Threats Decision expressly *requires* Member States and the Commission to consult each other in the HSC^24^ with a view to coordinating Member States’ public health responses and crisis communication.

It is manifest that neither occurred until approximately mid-March 2020, after the Italian government activated the EU civil protection mechanism established under the “solidarity clause” foreseen in Article 222 TFEU.

Yet, on 25 January 2020, the ECDC alerted all Member States that:

In light of the currently available information … the potential impact of 2019-nCoV outbreaks is high and further global spread is likely.^25^

Member States failed to come together and enacted instead their national risk responses. Several factors explain the ineffectiveness of the EU Cross-border Health Threats mechanism as currently designed and operationalised,^26^ but they essentially all point to a major structural cause.

To ensure that EU-wide public health coordination could effectively happen presupposes the existence of a minimum level of coordination among Member States’ healthcare exclusive competence. Thus, for instance, EU public health emergency coordination presupposes the existence of common methods for data collection on the spread of the virus, the characteristics of infected and recovered persons and their potential direct contacts – an EU-wide common testing strategy for cross-border cooperation in healthcare emergency assistance. Virtually all of these areas fall under the exclusive healthcare competence of each Member State. The EU not only has none of these frameworks in place, but – as dramatically showed by COVID-19 – it continues as well to lack a mapping of its Member States’ emergency preparedness plans.^27^

## III. Phase 2: from regulatory emulation to regulatory coordination?

After weeks of strictly enforced social distancing measures across the European continent, it became clear that a policy of indiscriminate social distancing could not last indefinitely: while these restrictive measures seemed necessary to slow down the spread of the virus and have saved tens of thousands of lives, they came at high social and economic costs.

Although justified in the beginning under a cost–benefit analysis performed during the initial emergency – showing that the benefits from strict social distancing in terms of lives saved significantly outweigh the economic costs^28^ – their justification weakened over time, due to the gradual manifestation of major economic, social and distributive costs.^29^ As our knowledge of the virus and the disease evolves, as well as that of the effects of different risk suppression measures, a continuous assessment on whether national responses are still proportionate is needed. If different national risk responses inevitably produce cross-border effects, these are in turn amplified when these measures are lifted – or fail to be reintroduced – as the epidemiological situation evolves.

This explains why the EU stepped in when Member States moved to phase 2 of the COVID-19 crisis and lifted some of their restrictions.^30^ This greater involvement is not only required in order to eventually discharge its Treaty mandate – that of pursuing a high level of health protection across all policy areas – but also to ultimately save lives.

If the uncoordinated national responses during phase 1 eventually converged on a fundamentally common approach of social distancing, thus contributing to slowing down the spread of the virus while saving tens of thousands of lives, an uncoordinated exit approach may lead to the opposite outcome.

As the emergency phase winds down, Member States find themselves incentivised to experiment with new “regulatory mixes” by lifting some restrictions and introducing new risk management measures. A good example is offered by countries such as Austria and Czechia that have accompanied the lifting of some major restrictions with new requirements such as the use of masks, be it in shops, on public transport or in any public space.

This calls for an EU-wide coordinated approach to the lifting of restrictive measures (and potentially their reintroduction) over the next phases of the pandemic and requires an adequate enforcement. In response to the European Council Members’ call for an exit strategy that is coordinated with Member States, the EU Commission and the Council itself prepared a Joint European Roadmap towards lifting COVID-19 containment measures (hereinafter “EU Exit Roadmap”). This document takes into account how the specific epidemiological situation, territorial organisation, healthcare service arrangements, population distributions or economic dynamics might affect Member States’ decisions on where, when and how measures are lifted.

The EU Exit Roadmap offers three main criteria to assess whether the time has come to begin to relax the confinement for each and every Member State^31^:

- *An epidemiological criterion* showing that the spread of the disease has significantly decreased for a sustained period of time;
- *Sufficient health system capacity* (ie the extent to which the different healthcare systems can cope with future increases in infection rates after lifting of the measures);
- *Appropriate monitoring capacity*, including large-scale testing capacity to detect and monitor the spread of the virus combined with contact tracing and quarantine capacity in case of the reappearance and further spread of infections.

This rather unusual guidance document strikes a fine balance between the need for EU-wide coordination and Member States’ different country-specific needs and cost–benefit calculus. It essentially introduces a set of meta-criteria or benchmarks framing the exercise of Member States’ public health prerogatives. In so doing, it also leaves each Member State the choice, depending on their size and organisation, regarding “what level of compliance with the criteria above should be assessed” (eg the regional or macro-regional level rather than at the national level).

This roadmap, together with a flurry of new documents freshly produced under time pressures by the EU Commission through its above-described public health emergencies bodies, represents an attempt at internalising the cross-border effects that are inherent to any national risk response in a highly integrated and interdependent EU. These guidance documents include the “COVID-19 Guidelines for Border Management Measures to Protect Health and Ensure the Availability of Goods and Essential Services”,^32^ the “Guidelines on EU Emergency Assistance in Cross-Border Cooperation in Healthcare related to the COVID-19 crisis”,^33^ the “Recommendation App on Contact Tracing (The Toolbox)”^34^ and the proposed “Guidance for Common Testing Strategies”. While adopted in a situation of emergency, these guidance documents show a timid yet auspicious attempt by the EU to operationalise untested competences contained in the Treaties and to do so in a situation of emergency.

The question is then if and to what extent the Commission will be invoking those guidelines – and ensuring the compliance with them – notably the EU Exit Roadmap, in the next stages of the COVID-19 crisis.

Due to the intertwined application of both positive and negative integration provisions, the EU – notably the EU Commission – is called upon to discharge its duty to balance public health benefits against other social and economic impacts, and to do so within the limits of the EU-conferred competences.

What if a given Member State lifts its COVID-19 restrictions too early (ie in the absence of a “significant decrease of the spread of the disease for a sustained period of time”)? What if it does so despite not having “sufficient health capacity” in case of the reappearance and further spread of the infection? Or what if a country fails to reintroduce restrictions when the spread of the virus has significantly increased?

Far from constituting EU legal acts under the meaning of Article 289 TFEU and producing legal effects invocable by third parties, these guidelines are set to raise legitimate expectations vis-à-vis EU citizens, companies and also Member States. While they may certainly be used to contextualise the examination of the legality of these national measures under EU law, in particular their proportionality, it remains unclear what their most immediate and long-term legal implications may be. Theoretically, should they go through the legislative process and be transformed into legislative acts, the EU COVID-19-related guidelines could qualify as “incentive measures” under the new and untested Article 168(5). Incentives measures would emerge as a novel tertium genus falling in between existing coordination public health measures and prohibited harmonisation of public health measures.

Be that as it may, by testing the outer limits of the EU public health competence, COVID-19 is set to go down in history as a major catalyst in the advancement of EU health emergency action.

In the meantime, COVID-19 is a painful, tangible reminder that EU coordination competences do matter. Indeed, legally defining – or redefining – *who* does *what*, *how* and *when* under EU law is a matter of life or death as never before across the continent.

## Footnotes

1 On 25 January, the European Centre for Disease Prevention reported: “In light of the currently available information, ECDC considers that the potential impact of 2019-nCoV outbreaks is high and further global spread is likely”, available at <https://www.ecdc.europa.eu/en/publications-data/communicable-disease-threats-report-19-25-january-2020-week-4>.

2 Decision No 1082/2013/EU of the European Parliament and of the Council of 22 October 2013 on serious cross-border threats to health and repealing Decision No 2119/98/EC, OJ L 293, 5.11.2013, pp 1–15.

3 On this point, see, in this special issue, eg, K Purnhagen, A de Ruijter, ML Flear, TK Hervey and A Herwig, “More Competences than You Knew? The Web of Health Competence for Union Action in Response to the COVID-19 Outbreak” (2020) European Journal of Risk Regulation doi:10.1017/err2020.35. For an examination of the EU public health competence beyond Art 168 TFEU, see also A de Ruijter, *EU Health Law & Policy: The Expansion of EU Power in Public Health and Health Care* (Oxford, Oxford University Press 2019); A Alemanno and A Garde, “The Emergence of an EU Lifestyle Policy: The Case of Alcohol, Tobacco and Unhealthy Diets” (2013) 50(6) Common Market Law Review 1745; A Alemanno and A Garde, “Regulating Lifestyles in Europe: How to Prevent and Control Non-Communicable Diseases Associated with Tobacco, Alcohol and Unhealthy Diets?” (2013) 7 Swedish Institute for European Policy Studies <http://www.sieps.se/en/publications/2013/regulating-lifestyles-in-europe-how-to-prevent-and-control-non-communicable-diseases-associated-with-tobacco-alcohol-and-unhealthy-diets-20137>.

4 Officials believe Italy’s Patient Zero, the first person to have brought COVID-19 to the country, is likely a German who travelled to northern Italy around 25 January 2020; see, eg, R Donadio, “I Can’t Stop Thinking About Patient One”, *The Atlantic*, 16 April 2020.

5 Although the specifics of a pandemic are unknowable, a variety of sources have predicted over the years a pandemic presenting the features of COVID-19. Most recently, a 2019 report by the Global Preparedness Monitoring Board, published under the auspices of the World Health Organization (WHO), made a compelling case for preparedness: “The world is at acute risk for devastating regional or global disease epidemics or pandemics that not only cause loss of life but upend economies and create social chaos”; “A World at Risk: Annual Report on Global Preparedness for Health Emergencies”, September 2019.

6 See also, eg, the WHO published its first Disease Outbreak News on the new virus on 5 January 2020. It contained a risk assessment and advice and reported on what China had told the WHO about the status of patients and the public health response on the cluster of pneumonia cases in Wuhan: <https://www.who.int/csr/don/05-january-2020-pneumonia-of-unkown-cause-china/en>. The ECDC reported the following on 25 January: “In light of the currently available information, ECDC considers that the potential impact of 2019-nCoV outbreaks is high and further global spread is likely”.

7 On the Swedish approach and its major sanitary and legal implications, see E Herlin-Karnell, “Corona and the Absence of a Real Constitutional Debate in Sweden”, *VerfBlog*, 10 April 2020 <https://verfassungsblog.de/corona-and-the-absence-of-a-real-constitutional-debate-in-sweden>; “Why Swedes Are Not Yet Locked Down”, *The Economist*, 4 April 2020.

8 See NM Ferguson et al, “Impact of Non-Pharmaceutical Interventions (NPIs) to Reduce COVID-19 Mortality and Healthcare Demand”, Imperial College London, 16 March 2020 <https://doi.org/10.25561/77482>.

9 The ultimate aim of suppression is to reduce the reproduction number (the average number of secondary cases each case generates), R, to below 1, and hence to reduce case numbers to low levels or (as for SARS or Ebola) eliminate human-to-human transmission.

10 Although to slightly different degrees, the only EU Member States that have adopted such a draconian measure are Italy, Spain and France. See European Commission, “Governmental Measure Data Base” <https://webcritech.jrc.ec.europa.eu/covidSt>.

11 D Thym, “Travel Bans in Europe: A Legal Appraisal,” *VerfBlog*, 19 March 2020 <https://verfassungsblog.de/travel-bans-in-europe-a-legal-appraisal>.

12 While only a few countries have mandated the use of masks by the general population (eg Czechia, Austria, etc.), the unfolding of the COVID-19 outbreak has considerably accelerated the number of countries recommending their use. At the time of writing, the pan-European guidance by the ECDC states: “The use of face masks in the community should be considered only as a *complementary* measure and not as a replacement for established preventive measures, for example physical distancing, respiratory etiquette, meticulous hand hygiene and avoiding touching the face, nose, eyes and mouth”; see European Centre for Disease Prevention and Control, *Using Face Masks in the Community* (Stockholm, ECDC 2020).

13 As examined in the *Verfassungsblog*’s “Fighting COVID 19” debate <https://verfassungsblog.de/category/debates/fighting-covid-19-debates>.

14 I Krastev, “Copycat Coronavirus Policies Will Soon Come to an End”, *Financial Times*, 7 April 2020.

15 For an initial attempt at theorising on the concept of emergency risk regulation, see A Alemanno (ed.), *Governing Disasters: The Challenges of Emergency Risk Regulation* (Cheltenham, Edward Elgar 2011).

16 European Commission, COVID-19 Guidelines for border management measures to protect health and ensure the availability of goods and essential services, Brussels, 16.3.2020 C(2020) 1753 final.

17 <https://www.europarl.europa.eu/factsheets/en/sheet/49/public-health>.

18 On this point, see, in this Special Issue, Purnhagen et al, supra, note 3.

19 Decision No 2119/98/EC of the European Parliament and of the Council of 24 September 1998 setting up a network for the epidemiological surveillance and control of communicable diseases in the Community, OJ L 268, 3.10.1998, pp 1–7.

20 Decision No 1082/2013/EU of the European Parliament and of the Council of 22 October 2013 on serious cross-border threats to health and repealing Decision No 2119/98/EC Text with EEA relevance, OJ L 293, 5.11.2013, pp 1–15.

21 <https://ec.europa.eu/health/sites/health/files/preparedness_response/docs/decision_serious_crossborder_threats_22102013_en.pdf>.

22 European Commission Decision of 16.3.2020 setting up the Commission’s advisory panel on COVID-19, Brussels, 16.3.2020 C(2020) 1799 final.

23 Under Art 9 of the Cross-border Health Threats Decision, national competent authorities or the Commission shall notify an alert in the Early Warning Response System where the emergence or development of a serious cross-border threat to health fulfils the following criteria: (1) it is unusual or unexpected for the given place and time, or it causes or may cause significant morbidity or mortality in humans, or it grows rapidly or may grow rapidly in scale, or it exceeds or may exceed national response capacity; (2) it affects or may affect more than one Member State; and (3) it requires or may require a coordinated response at the Union level.

24 Art 11(1) of Decision No 1082/2013/EU of the European Parliament and of the Council of 22 October 2013 on serious cross-border threats to health and repealing Decision No 2119/98/EC Text with EEA relevance, OJ L 293, 5.11.2013, pp 1–15.

25 ECDC, “Communicable Disease Threats Report, 19–25 January 2020, week 4” <https://www.ecdc.europa.eu/en/publications-data/communicable-disease-threats-report-19-25-january-2020-week-4>.

26 For a prescient and timely overview, see ML Flear and A de Ruijter (eds), “Symposium on European Union Governance of Health Crisis and Disaster Management” (2019) 10(4) European Journal of Risk Regulation.

27 EM Speakman, S Burris and R Coker, “Pandemic Legislation in the European Union: Fit for Purpose? The Need for a Systematic Comparison of National Laws” (2017) 121(10) Health Policy 1021.

28 For a US study, see M Greenstone and V Nigam, “Does Social Distancing Matter? (30 March 2020). University of Chicago, Becker Friedman Institute for Economics Working Paper No. 2020-26. Available at SSRN: <https://ssrn.com/abstract=3561244>. In Europe, no cost–benefit analysis was publicly made available at the time of writing, neither at the national nor at the EU level.

29 For a critique of the standard cost–benefit analysis of COVID-19, see M Adler and J Hammit, “A Better Way to Grapple with Benefit–Cost Trade-Offs in a Pandemic”, *The Hill*, 13 April 2020.

30 See “Joint European Roadmap towards Lifting COVID-19 Containment Measures”, April 2020, available at <https://ec.europa.eu/info/sites/info/files/communication_-_a_european_roadmap_to_lifting_coronavirus_containment_measures_0.pdf>.

31
ibid.

32 Brussels, 16.3.2020 C(2020) 1753 final.

33 Brussels, 3.4.2020 C(2020) 2153 final.

34 <https://ec.europa.eu/info/files/recommendation-apps-contact-tracing_en>.

